# Prevalence and factors determining psychoactive substance (PAS) use among Hawassa University (HU) undergraduate students, Hawassa Ethiopia

**DOI:** 10.1186/1471-2458-14-1044

**Published:** 2014-10-07

**Authors:** Andargachew Kassa, Fiker Taddesse, Aweke Yilma

**Affiliations:** HUCMHS, Hawassa University, Po Box 1560, Hawassa, Ethiopia

**Keywords:** Psychoactive substance, University students, Khat, Alcohol, Cigarettes, Marijuana

## Abstract

**Background:**

Use of psychoactive substances (PAS) early in school age implies drug dependence in later life. Although no studies have been conducted on undergraduate students of Hawassa University, a few studies in Ethiopia have reported that alcohol, khat, and cigarette are the commonly abused PASs among young high school/undergraduate university students. Therefore, this study was designed to establish the prevalence of and predictors for PAS use among undergraduate HU Students.

**Methods:**

An institution-based quantitative cross-sectional study using the self-administered WHO Model Core Questionnaire to collect information on use of various Psychoactive Substances (PASs) was conducted from June to July 2011. A multistage stratified sampling method was employed to select a total of 586 undergraduate HU students as study participants. Bivariate and multivariate logistic regression analysis were done to determine factors affecting PAS use.

**Results:**

Lifetime, past 12 months, and current prevalence rate for overall PAS were 53.6%, 45.7%, and 35.5% respectively. The study depicted that in the past 12 months of the study period 40.8% used alcohol, 20.3% chewed khat, 11.9% smoked cigarettes, and 0.9% used marijuana. The prevalence of other illicit PASs such as Ecstasy, lysergic diethylamide (LSD), cocaine, crack, heroin, solvents or inhalants and un-prescribed psycho active medications was found zero percent (0%). Having family members who used PAS, peer influence, being male, and living alone during school age were found to be positively associated with overall PAS use in the past 12 months.

**Conclusion:**

The prevalence of PAS use among undergraduate HU students is high. Designing effective strategies to reduce PAS use should be everyone’s priority.

## Background

Psychoactive Substance (PAS) use is becoming commonly known for compromising the health and resulting in the death of millions of individuals every year [1–3]. PAS’s include licit, illicit, and prescribed psychoactive medications. Alcohol, cigarette, and khat are among the licit and controlled drugs, while marijuana, cocaine, heroin, lysergic diethylamide (LSD), crack, and Ecstasy are illicit drugs [4, 5]. Abuse of prescription stimulants like amphetamines, benzodiazepines, hallucinogens, ketamines, and nonmedical use of prescription stimulants (NPS) are also commonly reported [4]. Compared to other deadly communicable diseases, PAS use is among the leading cause of death [2–5]. Repeated use of PAS is linked not only to disease and death but also to addiction, dependence [2, 6, 7], and predisposition for criminal and antisocial behaviors. When young people are at such risk, such situation demands receive global attention [8, 9].

Some studies from Ethiopia and neighboring counties have investigated the magnitude and pattern of PAS use among university students. 84% of students were using alcohol at one Kenya private university [10]. Khat is one of the most commonly abused PAS in countries like Ethiopia, Yemen, Kenya, Uganda, and other Arabic countries. For instance, lifetime prevalence of khat use is reported to be 28.4% among Axum University students of Ethiopia [11] and 23.1% for university students of Jazan region, Saudi Arabia [12]. One study reported that 2.8% of university students in Eldoret Kenya used Marijuana [13]. There are also other surveys which are done at Nigeria, Central America, Europe, Australia, and Dominican Republic depicting the use of other illicit drugs and NPS [14–18].

Certain factors are known to influence the use of PAS: age, male gender, religion, having family members who use PAS, having friends who use PAS (ie, peer influence), level of education, level of maternal and paternal education, monthly pocket money, ethnic background, and life style during school age (eg, living with family, living alone, or being orphaned) [11, 12, 19, 20]. Understanding the nature and magnitude of substance use as well as the factors that contribute to it should allow the design of effective intervention strategies. However, the pattern and magnitude of factors affecting PAS use, especially the use of most of the illicit drugs and NPS among Ethiopian university students, is not well known. Hence, this study was designed to determine the prevalence of PAS use and the factors that influence it among undergraduate students of Hawassa University (HU).

## Methods

This study was conducted from June to July 2011 to assess the magnitude and factors determining PAS use among undergraduate students of HU, Ethiopia. Four campuses were involved: The first three are campuses of HU: Main campus, Referral campus, and Agricultural campus. All are located in Hawassa, the capital city of the Southern Nations Nationalities and Peoples Region, 275 km far from Addis Ababa, the Capital city of Ethiopia. The fourth campus, Wondogenet Forestry College, is located at Wondogenet town of Sidama Zone. During the study period, the University had a total of 15,104 undergraduate students enrolled in 58 academic departments. All undergraduate students came from all regions of Ethiopia.

The study used an institution-based quantitative cross-sectional study design. The source population included all regular undergraduate students attending the four colleges of Hawassa University. Excluded from the study were all who were seriously ill and absent during the study period. Using a single-population proportion formula, the sample size was calculated based on the following assumptions: An assumption of 22.0% past 12 month PAS use prevalence was used based on research conducted among undergraduate medical students of Addis Ababa University in Ethiopia [21]. Also assumed was a 5% margin of error (precision) at 95% confidence level (CI). With a nonresponse rate of 12% and a design effect of two for multistage sampling, the total sample size was computed to be 590. The study participants were selected using a multistage stratified sampling technique. During the first stage, by using simple random sampling technique, twenty five (25) departments were selected from a total of 58 departments. In the second stage, departments in each field of study were further stratified by years of study and sex, on the assumption that duration of stay in the campus and sex would affect PAS use. The total sample size was distributed proportionally to the selected departments based on total number of students in each year of study according to sex. Finally, individual students fulfilling the inclusion criterion were randomly selected to be study participants.

A pretested, structured, self-administered questionnaire, the World health Organization (WHO) Model Students’ Substance Use Core Questionnaire, was used for data collection. Information was collected on the use of various PASs, including alcohol, tobacco, marijuana, LSD, Ecstasy, cocaine, heroin, and other psychoactive NPS [22]. This questionnaire also gathers data on socio-demographic aspects of students, frequency of PAS use, accessibility of PAS, and perceived health effects of PAS. The questionnaire has also questions designed to identify the type of problems a student encountered as a result of PAS use; the study participant had 14 answers from which to choose. Some drug-related questions in the WHO Model Students’ Substance Use Questionnaire involved the nonexistent drug “Relevin” included as a “validity check” to ensure that students did not overestimate their drug use [22]. This instrument has been standardized and used in many different countries to collect data on PAS use among youths [13, 23, 24]. An additional question to determine student’s reason for using PAS was prepared by reviewing relevant literatures. This was a question that asked about students’ reasons for using PAS. Possible answers included eight suggested reasons [13, 23, 24]. The questionnaire was customized to fit the context and objectives of the current study and was also meticulously translated to Amharic so that the students could understand the questions. A pre- test was performed on 10% of the sampled subjects at Dilla University.

Questionnaires were distributed by trained data collectors to students who attended class on the day of distribution. The data collectors provided a brief introduction to the study, explaining the method of data collection and instructing the students to avoid writing their names on the questionnaires. The study was conducted in the absence of teachers.

Some of the terminologies used in this study were defined as follows:

- “Lifetime PAS use” was defined as respondents who admitted to having ever used at least one of the PASs listed in the questionnaire.
- “Past twelve Month PAS use” was defined as students who use PASs listed in the questionnaire in the 12 months prior to the date of data collection.
- “Current prevalence of PAS use” was defined as the proportion of students who used PAS within 30 days preceding the study.

The collected data were cleaned, coded, entered, and analyzed using SPSS computer software package version 20. Descriptive and summary statistics of sociodemographic variables were presented using, proportion, mean median, range, standard deviation, and frequency tables. We used Binary Bivariate and Multivariate Logistic Regression analysis. Bivariate analysis was done, and variables with p-value less than 0.2 were included in the multiple logistic regression analysis. Odds Ratio (OR) and 95% confidence intervals were also computed along with the corresponding p-value.

Ethical clearance for the study was obtained from Institutional Review Board of College of Medicine and Health Sciences, HU. To ensure voluntary participation of each participant, a written and signed informed consent was obtained from each student. Additionally, the confidentiality of the information was ensured by using anonymous questionnaires and by maintenance of the data in a safe and protected place.

## Results

Out of 590 students who participated in the study, 586 completed the questionnaires (response rate, 99.3%). Among the study subjects, 479 (81.7%) were male, and 107 (18.3%) were female. The majority of students or 468 (69.2%) were 20–24 years of age, and the mean age of the respondents was 20.7 years (SD ± 1.49 years). Three hundred fifty (59.7%) of the respondents were Orthodox Christians, and 186 (31.7%) were Amhara in Ethnic group (Table 1).

**Table 1:** **Socio-demographic characteristics of Hawassa University Students by sex, (n = 586) June/July 2011**

Characteristics	Sex		Total n (%)
	Male n (%)	Female n (%)	
**Age group(years)**			
15-19	72(12.3% )	34(5.8%)	106(18.1%)
20-24	397(67.8%)	71(12.1%)	468(79.9%)
25-30	10(1.7%)	2(0.3%)	12(2.0%)
**Total**	**479 (81.8%** **)**	**107(18.2%** **)**	**586(100%** **)**
**Religion**			
Orthodox Christian	285 (48.6%)	65(11.1%)	350(59.7%)
Protestant Christian	127(21.7%)	29(4.9%)	156(26.6%)
Muslim	53(9.0%)	9(1.5%)	62(10.6%)
Others	14(2.4%)	4(0.7%)	18(3.1%)
**Total**	**479(81.7%** **)**	**107(18.3%** **)**	**586(100%** **)**
**Year of education**			
Year I	195(33.2%)	48(8.2%)	243(41.3%)
Year II	115(19.6%)	30(5.1%)	145(24.7%)
Year III	148(25.2%)	23(3.9%)	171(29.1%)
Year IV	13(2.2%)	3(0.5%)	16(2.7%)
Year V	5(0.9%)	2(0.3%)	7(1.2%)
Year V I/internship	3(0.5%)	1(0.2%)	4(0.7%)
**Total**	**479(81.4%** **)**	**107(18.2%** **)**	**586(100)**
**Mother’s educational level**			
Illiterate	192(32.6%)	14(2.4%)	206(35.2%)
Elementary to grade 12	202(34.3%)	54(9.2%)	256(43.5%)
College or University	69(11.7%)	38(6.5%)	107(18.2%0
Do not Know	16(2.7%)	1(0.2%)	17(2.9%)
**Total**	**479(81.4%** **)**	**107(18.2%** **)**	**586(100%** **)**
**Father’s educational level**			
Illiterate	118(20.1%)	8(1.4%)	126(21.5%)
Elementary to grade 12	228(38.9%)	44(7.5%)	272(46.4%)
College or University	114(19.5%)	52(8.9%)	166(28.3%)
Do not Know	19(3.2%)	3(0.5%)	22(3.8%)
**Total**	**479(81.7%** **)**	**107(18.3%** **)**	**586(100%** **)**

The overall lifetime and past 12-month prevalence of PAS use among the study subjects were 315 (53.6%) and 269 (45.7%), respectively. The magnitude (prevalence) of PAS use in the past 12 months was 40.8% for alcohol, 20.3% for khat, 11.9% for cigarette, and 0.9% for marijuana. Fifty-two (9.0%) of the study participants reported that they abused shisha or hookah smoke in their lifetime. The rate of poly drug use (two or more PASs) was 18.3%. The rate of use of other PASs (illicit, licit, or NPS medications such as amphetamine, benzodiazepines, hallucinogens, ketamine, LSD, Ecstasy, heroin, cocaine, and crack) was zero (0%) (See Table 2).

**Table 2:** **The life time, last 12 month & current prevalence of psychoactive substance use among Hawassa University Undergraduate Students (n = 586) June/July 2011**

Type of substances used	Total (n = 586)					
	No n = 586	%	Male		Female	
			No	%	No	%
1. Used PAS ever	315	53.6%	278	88.1	37	11.7
2. Used PAS in the past 12 months	269	45.7%	239	88.7	30	11.1
3. Used PAS in the past 30 days	209	35.5	183	87.5	26	12.4
4. Ever polydrug use	142	24.2%	130	91.6	12	8.4
5. Poly drug use in the past 12 months	106	18.1%	94	88.7	12	11.3
6. Current poly drug use	83	14.2%	72	86.8	11	13.3

Most students (79.4%) replied that they had heard about amphetamines and other prescription medical stimulants. Half (51.9%) reported that they had information about marijuana (hashish). Study participants were least familiar with ecstasy.

The mean ages at which the respondents started drinking alcohol, chewing khat, and smoking cigarettes were 15.4 years (SD ± 3.5 years), 16.6 years (SD ± 2.7 years), and 17.0 (SD ± 2.9 years), respectively. Of the 315 students who ever used PAS, 282 (89.5%) were able to remember the first substance they ever used in life. More than half (59.0%) reported that alchol was their gateway drug, followed by khat (20%).

Generally, students reported eight reasons for using PAS. ‘Peer influence’ and ‘to get energized for study’ were the most frequent reasons reported by 29.6% and 19.7% respondents respectively (See Table 3). Respondents reported all possible consequences of PAS use listed in the questionnaire. The most frequently reported consequence was ‘quarrel or argument” 19.3% (31 of 161 students), followed by “Scuffle or fight” 13.0% (21 of 161students). Regarding risky sexual behavior, 11 of 161 study participants (06.8%) reported that they had engaged in sex they regretted the next day and 9 of 150 respondents (6.0%) reported that they had engaged in unprotected sex due to their PAS use.

**Table 3:** **Reported reasons for psychoactive substance (PAS) use by Hawassa University Undergraduate Students (n = 315) June/July 2011**

S.N	Reasons for using substances	Frequency (n = 315)	Percent (%)
1.	Peer influence	90	29.6%
2.	To get energized to study	62	19.7%
3.	To avoid stress	47	14.9%
4.	To avoid sleep	46	14.6%
5.	To get happiness or pleasure	45	14.3%
6.	For testing	36	11.4%
7.	For stomach ailments	15	04.8%
8.	Family or keen members influence	10	03.1%

Socio-demographic and behavioral correlates assumed to be associated with PAS among the study participants were assessed using logistic regression (Table 4). Compared to being female, being male was a factor that increased the likelihood of PAS use within the last 12 months by more about three-fold (adjusted OR = 2.41, 95% CI = 1.5-3.9). Study participants who reported family use of PAS were more likely to use PAS in the past year as compared with those whose family didn’t use PAS (adjusted OR = 2.3, 95% CI = 1.4-3.8). Similarly, having a friend who used PAS increased the odds of PAS use two times higher than for those who didn’t have friends who used PAS (adjusted OR = 2.2, 95% CI = 1.3-3.6). Most importantly, living alone during school age increased the risk of PAS use by ten-fold, as compared to students who lived with their families (adjusted OR = 10.7, 95% CI = 1.3-82.9).

**Table 4:** **Bivariate and multivariate logistic regression analysis showing socio-demographic and behavioral correlates of psychoactive substance (PAS) use within the last 12 months among Hawassa University Undergraduate Students, June/July 2011**

Variables	Used PASU		COR (95% CI)	AOR (95% CI)
	Yes	No		
**Sex**				
**Male**	239	240	2.6 (1.8-3.7)	2.4 (1.5-3.9)
**Female**	30	77	1*	1*
**Family substance use history:**				
**Yes**	64	205	2.7 (1.7-4.2)	2.3 (1.4-3.8)
**No**	33	284	1*	1*
**Having friend who use PAS:**				
**Yes**	59	210	2.6 (1.6-4.2)	2.2 (1.3-3.6)
**No**	31	286	1*	1*
**Lived alone during School age:**				
**Yes**	9	260	10.9 (1.4-86.0)	10.2 (1.2-82.8)
**No**	1	316	1*	1*
**Monthly pocket money below 150 Birr**				
**Yes**	66	94	0.8 (0.5-1.1)	0.7 (0.5-1.0)
**No**	203	223	1*	1*
**Academic year two and above**				
**Yes**	174	169	1.6 (1.2-2.2)	1.4 (0.9-2.0)
**No**	148	95		
**Came from orphanage**			1*	1*
**Yes**	5	2	2.0 (0.6-15.5)	2.0 (0.3-12.1)
**No**	264	315	1*	1*

1* = reference.

## Discussion

This study reveals that PAS use among Hawassa University undergraduate students is high. The overall lifetime and current prevalence of PAS use among the study subjects were 53.6% (315) and 35.5% (209), respectively. In contrast to two other similar studies conducted among Ethiopian undergraduate university students of Axum [11] and Mekele [25], the lifetime prevalence was lower than that of reported for Mekele (82.7%) and somewhat higher than that of reported for Axum (45.9%). But the reported current prevalence of PAS use of Axum University students (44.8%), in contrast, is higher than HU students (35.5%). In contrast to two similar studies conducted at Eldoret Kenya [13] and Nigeria [23], both the lifetime and current prevalence of PAS findings among HU students were considerably lower: lifetime prevalence was 87.3% for Nigeria and 69.8% for Eldoret. The current prevalence of PAS for Nigeria was reported as 69.8%. This difference may be due to the differences in the years the studies were conducted, culture and policies of the respective universities, availability of substances, surrounding community culture, and how well the study participants understood the health risks of PAS.

The lifetime prevalence of polydrug (two or more PASs) use among the study participants was 24.2%. This rate is lower than that reported by a similar study conducted at Mekele University students, which showed a 45% use [25]. However, similar to the findings of this study is that both studies reported alcohol and khat as the most commonly used drugs, affirming the fact that these substances are gateway drugs for PAS student users.

Our study found that HU undergraduate students used only alcohol, khat, cigarettes, and marijuana. As compared to the results of other studies conducted in Ethiopian Universities, these are the most commonly used PASs [11, 19, 21, 25]. This is may be due to the fact that homemade alcoholic drinks are traditionally and socially acceptable for the vast majority of Ethiopian people. Availability of khat in every corner of Ethiopia due to its ever increasing cultivation by the rural farmers and its social acceptability probably accounts for the reason that it is the second most utilized PAS by HU students.

The investigators of this research observed that, in contrast to alcohol and khat, the price of cigarettes is lower than that of alcohol and khat. Students can also obtain cigarettes in the front main gates of all campuses. Nevertheless, similar to the findings of other studies [11, 21, 25], cigarette smoking is the third common PAS used by HU students. This may be due to the fact that the prevalence of cigarette smoking is also lower in the general population of Ethiopia.

The mean age at which HU students started PAS was 15.4 years (SD ± 3.5 years) for alcohol, 16.6 years (SD ± 2.7 years) for khat, and 17.0 (SD ± 2.9 years) for cigarettes, which demonstrates the importance of designing and implementing intervention policies for PAS use in elementary school and continuing this process through college/university years.

A well-established link exists between PAS use and causal and unprotected sex [26, 27], which in turn leads to higher risks of HIV/AIDS, other STIs, and unplanned/teenage pregnancy and abortions [28]. In keeping with this finding, 161 respondents who used PAS, 11(6.8%) reported that they engaged in sex as a result of PAS use, and 9 of 150 (6.0%) engaged in unprotected sex. These data show that PAS use impairs judgment, leading to risky sexual behavior that in turn fuels the epidemics of HIV, STIs, and teenage pregnancy [26, 29].

The prevalence of marijuana, one of the illicit drugs in Ethiopia, was 2.2% for lifetime and 0.9% and 0.3% for past 12-month and 30-day use, respectively. This finding is lower than that reported by other studies. For example, one metaanalysis conducted on “youthful drug involvement in Central America and the Dominican Republic” revealed that 8% to 9% of school-age youth had a change to use marijuana and about 4% to 5% youths have actually engaged in marijuana use on at least one occasion [15].

Some of the studies conducted among university students of Ethiopia didn’t report students’ marijuana use. But, compared to this study, one of the studies conducted among urban and rural high school students reported a nearly similar lifetime prevalence of marijuana (hashish) use: 1.0% & 2.7% respectively [30]. The prevalence in this study matched with what had been that found by a similar study conducted among university students in Kenya: reported life time hashish use was 2% [13]. The reason for low prevalence of hashish use among HU students may result from strong drug control and policing in Ethiopia, lack of availability of the drug, and the cost of the drug. However, this reported prevalence still testifies to the fact that this marijuana (hashish) continues to be cultivated on a small scale in the country in a way that is undetectable to authorities.

Most studies conducted in America, Europe, Australia, and Africa reported use of NPS (amphetamine, benzodiazepines, hallucinogens, and ketamine) and illicit drugs (LSD, Ecstasy, heroin, cocaine, and crack) [14–18]. However in our study we didn’t find use of NPS, the illicit drugs, and the non-existent drug Relevin. Compared to other similar studies conducted in Europe [31], America [16, 17], and other African countries [14, 18], this finding is astonishing. This may be explained by the fact that the majority of students didn’t know about most of the illicit substances or the availability of NPS, although most NPS were accessible. Another reason might be the strong police control over illicit drugs and the non accessibility of these illicit drugs. That seems why no one of the students reported the use of Relevin, intentionally included as a “validity check,” may indicate that study participants didn’t over estimate their substance use [22].

Being male (AOR = 2.4, 95% CI = 1.5-3.9), having PAS user friends (AOR = 2.17, 95% CI = 1.3-3.6), having family members who use PAS (adjusted OR = 2.3, 95% CI = 1.4-3.8), and living alone during school age (adjusted OR = 10.3, 95% CI = 1.3-82.9) were all factors that increased the odds of HU students using PAS within the past 12 months. These findings are consistent with reports from other similar studies [11, 13, 21, 23, 24, 29]. Reasons for the findings may include cultural influences that discourage females from using PAS, children’s and especially adolescents’ strong predisposition to learn from elder family members, parental modeling during early childhood, and peer influence during later childhood. In contrast, financial freedom and loose family control may be the very reasons for PAS use by school age students who live alone.

The limitation of the study is its cross-sectional study design, which resulted in an inability to establish trends and causality between PAS use and the potential risk factors. Also, the fact that data were self-reported could have led to recall bias and underreporting. Social desirability bias was another challenge, as was the possibility of purposeful exaggeration. Furthermore, generalization of the study findings beyond undergraduate university students may not be appropriate, and more studies may be warranted. To avoid social desirability bias, an anonymous questionnaire was used, and a secure, confidential environment was provided to ensure that students did not worry that they would be arrested for use of illicit drugs or face discrimination and stereotyping. Finally, the fact that the study was based on a reliable tool, the WHO student’s substance use questionnaire attests to its validity and reliability.

## Conclusions

The prevalence of PAS among undergraduate Hawassa University (HU) students is very high. PAS use by HU undergraduate students in descending order of frequency comprised alcohol, khat, cigarettes, and marijuana, the only illicit drug reported by HU undergraduate students. There were no reports of use of illicit drugs such as LSD, Ecstasy, heroin, cocaine, and crack or of NPS. Living alone during school age, being male, having family who used PAS, and having friends who used PAS, appear to be independent predictors of PAS use for undergraduate HU students.

Urgent but effective and well-designed substance (PAS) use prevention and health promotion programs should be designed by all concerned stake holders and students. Further studies should be conducted to determine the level of substance abuse and/or drug dependence and their ensuing health consequences. Such a study might be an ongoing, longitudinal study for monitoring the progress and outcomes of future interventions. Finally the importance of designing and implementing a cessation program, for those who already developed dependence on PAS, cannot be overemphasized.
